# The effect of colloid formulation on colloid osmotic pressure in horses with naturally occurring gastrointestinal disease

**DOI:** 10.1186/1746-6148-10-S1-S8

**Published:** 2014-07-07

**Authors:** Fausto Bellezzo, Timothy Kuhnmuench, Eileen S Hackett

**Affiliations:** 1 Department of Clincial Sciences, Colorado State University, Fort Collins, CO, 80523, USA

## Abstract

**Background:**

Naturally occurring gastrointestinal disease is an important cause of acute hypoproteinemia in adult horses and hydroxyethyl starch colloid fluid treatment is a component of supportive care in these cases to improve plasma volume and maintain colloid osmotic pressure (COP). The objectives of the present study were to compare 2 formulations of high molecular weight hydroxyethyl starch and their relative effect on COP, acid-base status, and survival of horses with acute hypoproteinemia secondary to gastrointestinal disease.

**Methods:**

Twenty adult horses, ≥ 1 year of age, were prospectively enrolled, with informed client consent, if they developed acute hypoproteinemia, defined as a plasma total protein <5.0 g/dL or albumin <2.2 g/dL during hospitalization while undergoing treatment for gastrointestinal disease. Horses were randomly assigned to receive a rapid infusion of either 6% hydroxyethyl starch in 0.9% saline or 6% hydroxyethyl starch in lactated ringers solution at a dose of 10ml/kg. Venous blood gas analysis, COP, and PCV were evaluated before and after colloid administration.

**Results:**

For both groups, average COP prior to treatment was 11.0 mmHg (9.7 – 12.2 mmHg) and post colloid treatment was 13.2 mmHg (12.0 -14.7 mmHg) [Normal range 18 – 22 mmHg]. COP was significantly increased with colloid treatment (p<0.001) but this increase was not significantly different between treatment groups. Venous pH did not change significantly with treatment. Twelve horses survived to hospital discharge and survival did not differ significantly between treatment groups.

**Conclusions:**

Post-treatment COP improved approximately 20% regardless of the formulation used, however, values did not reach the normal range of COP observed in healthy horses. Acid-base parameters were not significantly impacted by either treatment. Further study is needed to determine how these two products compare with regards to other outcome measures. Evaluation of the relative effects of colloid formulation in horses with clinical disease is a future area of interest.

## Background

Naturally occurring gastrointestinal disease is an important cause of acute hypoproteinemia in adult horses. Acute hypoproteinemia lowers colloid osmotic pressure (COP) and can result in negative sequellae, such as decreased circulating plasma volume, increased extracellular fluid accumulation, and impaired tissue oxygen delivery contributing to multiorgan failure [1,2]. Low COP increases risk of death in critical illness without intervention, therefore colloid fluid treatment is a component of supportive care in these cases [3]. Colloid fluids contain osmotically active macromolecules that are retained in the vasculature. Thus, colloids act to expand plasma volume by maintaining COP. Maintaining COP within normal ranges limits morbidity of disease by decreasing tissue edema and resultant organ dysfunction. Colloids used in horses include biologics such as plasma and whole blood, and synthetic colloids, such as hydroxyethyl starch, pentastarch, and dextrans [1]. Hydroxyethyl starch is the most commonly used colloid in adult horses [1].

Hydroxyethyl starches are commonly selected due to low cost, long shelf life, and avoidance of the use of biologic agents. Acidosis occurs following hydroxyethyl starch solution administration in people due to both dilution and hyperchloremia, and is less likely to occur when the starch diluent is lactated ringers versus saline [4,5]. Hydroxyethyl starch solutions are associated with increased mortality and severe renal injury when used in critically ill adult humans including those with sepsis, and excessive bleeding in humans undgergoing open heart surgery and cardiopulmonary bypass [6]. This has resulted in a recommendations for human health professionals to not use hydroxyethyl starch solutions in critically ill adults, patients with pre-existing renal dysfunction, patients with signs of renal injury, patients with coagulopathy associated with cardiopulmonary bypass, and patients with severe liver disease and to monitor renal and hepatic function testing following administration [6].

Artificial colloid formulation may impact outcomes due to efficacy or side effects, and this has not been evaluated in horses with clinical disease. Information on the anticipated effect of colloidal formulations on COP and survival is not currently available for veterinarians providing primary care to critical equine patients. To this end, a preliminary randomized prospective clinical trial was conducted to compare 2 formulations of high molecular weight hydroxyethyl starch and their relative effect on colloid osmotic pressure, acid-base status, in-hospital complications, and survival. We hypothesized that intravenous hydroxyethyl starch colloid supplementation would raise the COP in horses with low protein secondary to gastrointestinal disease, regardless of the formulation used. Further, we anticipated that hydroxyethyl starch in lactated ringers solution would be less commonly associated with acidosis than hydroxyethyl starch in saline solution.

## Methods

The experimental protocol was reviewed and approved by the Institutional Animal Care and Use Committee (#08-143A-01). Adult horses, ≥ 1 year of age, were prospectively enrolled, with informed client consent, if they developed acute hypoproteinemia, defined as a plasma total protein <5.0 g/dL measured by refractometry or albumin <2.2 g/dL measured by bromcresol green photometric method (Cobas 510 Chemistry Analyzer, Roche Diagnostics Corporation, Indianapolis, IN) during hospitalization while undergoing treatment for gastrointestinal disease. Signalment, primary clinical diagnosis, interval between hospital admission and randomization, and pre-treatment level of protein or albumin as treatment trigger were recorded. Horses were assigned at random to one of two treatment groups. Horses in group 1 (n=10) were treated with 10 mL/kg of 6% hydroxyethyl starch in 0.9% saline (Hetastarch, Abbott Laboratories, Chicago, IL, USA). Horses in group 2 (n=10) were treated with 10 mL/kg of 6% hydroxyethyl starch in lactated ringers solution (Hextend, Abbott Laboratories, Chicago, IL, USA). Both hydroxyethyl starch products had an average molecular weight of 670 kD, with a range of 450 to 800 kD, 0.75 molar substitution, and 1:20 degree of branching, with one α-1,6 branch for every 20 glucose monomer units. Colloid products were administered via jugular intravenous catheter with an infusion pump at 200 mL/minute and no other medications or intravenous fluid therapies were administered simultaneously. Colloid osmotic pressure (Wescor 4420 Colloid Osmometer, Salt Lake City, UT, USA), venous blood gas (ABL2000 Blood Gas Analyzer, Radiometer, Copenhagen, Denmark), and packed cell volume (PCV) were evaluated before and immediately following colloid administration. Treatments assigned by the primary clinician were unrestricted. Complications arising from the primary disease or from independent causes, as indicated by the clinician of record, that developed during hospitalization were recorded in all horses. Short term survival was defined as survival to hospital discharge.

All continuous variables underwent Shapiro-Wilk analysis for normality. As variables were normally distributed, continuous data were reported as mean and 95% confidence interval. A paired t test was used to evaluate changes in COP pre- and post-colloid administration in both groups. A two sample t test was used to assess differences between groups in COP change following colloid administration. Fisher’s Exact test was used to compare mortality and complications between groups. Level of significance for all comparisons was *P*<0.05. Analysis was conducted using commercially available software (StataCorp, College Station, TX, USA).

## Results

Treatment group 1 was composed of 5 geldings, 4 intact mares, and 1 ovariectomized mare, with 4 American Quarter Horses, 3 Thoroughbreds, and one each Arabian, American Paint Horse, and Friesian. Gastrointestinal disease diagnoses in treatment group 1 included primary colitis (n=7) and colitis following surgical correction of large colon volvulus (n=3), and mean interval between admission and colloid treatment was 23 hours. Treatment group 2 was composed of 4 geldings, 4 intact mares, and 2 stallions, with 4 American Quarter Horses and one each Arabian, American Paint Horse, Oldenburg, Standardbred, Thoroughbred, and mixed breed horse. Gastrointestinal disease diagnoses in treatment group 2 included primary colitis (n=3), colitis following surgical correction of large colon volvulus (n=4), proximal enteritis (1), and peritonitis secondary to perforating foreign body (n=1) or jejunal strangulation (n=1), and mean interval between admission and colloid treatment was 15 hours. Horses qualified for study enrolment based on plasma protein concentration (19) and plasma albumin concentration (18). Following randomization, age, weight, COP, plasma albumin, total protein, PCV, and blood gas analytes did not differ between the two treatment groups prior to colloid administration, as described in Table 1.

**Table 1:** Summary of selected clinical and clinicopathologic variables of horses with naturally occuring gastrointestinal disease within treatment groups prior to and following colloid administration. Results listed as mean and [95% Confidence Interval]. Significant differences (P<0.05) were not detected between treatment groups following randomization.

Variable [reference interval]	Group 1: hydroxyethyl starch in 0.9% saline solution	Group 2: hydroxyethyl starch in lactated ringers solution	*P* value for treatment group comparisons
Age (years)	8 [3.8 – 11.3]	12 [7.2 – 16.8]	*P*=0.118
Weight (kg)	496 [441 – 551]	518 [433 – 604]	*P*=0.627
Pre-treatment albumin [25-35 g/L]	18 [16 – 19]	19 [16 – 23]	*P*=0.419
Pre-treatment total protein [50-78 g/L]	42 [39 – 46]	41 [38 – 44]	*P*=0.645
Pre-treatment COP [18-22 mmHg]	11.0 [9.4 – 12.5]	11.0 [8.6 – 13.5]	*P*=0.945
Post-treatment COP [18-22 mmHg]	13.8 [12.2 – 15.4]	12.9 [10.4 – 15.4]	*P*=0.512
Pre-treatment venous pH [7.38 – 7.44]	7.38 [7.33 – 7.44]	7.41 [7.38 – 7.43]	*P*=0.442
Post-treatment venous pH [7.38 – 7.44]	7.38 [7.32 – 7.43]	7.40 [7.37 – 7.43]	*P*=0.435
Pre-treatment venous sodium [130–142 mmol/L]	132 [127 – 137]	136 [134 – 138]	*P*=0.119
Post-treatment venous sodium [130–142 mmol/L]	134 [128 – 140]	136 [135 – 138]	*P*=0.383
Pre-treatment chloride [96–110 mmol/L]	99 [96 – 102]	102 [100 – 105]	*P*=0.098
Post-treatment chloride [96–110 mmol/L]	102 [98 – 105]	104 [101 – 107]	*P*=0.344
Pre-treatment bicarbonate [22–30 mmol/L]	24 [21 – 28]	26 [23 – 30]	*P*=0.322
Post-treatment bicarbonate [22–30 mmol/L]	23 [19 – 28]	27 [24 – 30]	*P*=0.155
Pre-treatment anion gap [10–17 mmol/L]	12 [10 – 14]	10 [9 – 12]	*P*=0.200
Post-treatment anion gap [10–17 mmol/L]	11 [9 – 13]	9 [8 – 11]	*P*=0.114
Pre-treatment venous lactate [1.1 – 1.8 mmol/L]	2.3 [1.1 – 3.5]	1.6 [1.0 – 2.1]	*P*=0.204
Post-treatment venous lactate [1.1 – 1.8 mmol/L]	2.1 [1.0 – 3.3]	1.5 [1.2 – 1.9]	*P*=0.275

Adverse reactions were not observed with either colloid treatment. When treatment groups were combined, mean COP prior to administration was 11.0 mmHg [9.7 – 12.2 mmHg] and post colloid treatment was 13.2 mmHg [12.0-14.7 mmHg]. Clinicopathologic effects of colloid administration are described by treatment group in Table 1. COP was significantly increased with colloid treatment (*P*<0.001) regardless of treatment group. This COP increase was not significantly different between treatment groups (*P*=0.051). Venous pH did not change significantly with colloid treatment (*P*=0.226) and did not differ between groups (*P*=0.866).

During remaining hospitalization, 1 horse in group 1 (hydroxyethyl starch in normal saline solution) and 3 horses in group 2 (hydroxyethyl starch in lactated ringers solution) were treated with an additional 10 mL/kg dose of hydroxyethyl starch in normal saline solution for persistent signs associated with hypoproteinemia. In addition, 2 horses in each treatment group were treated with 4 – 10 mL/kg of intravenous commercial plasma.

Five of 10 horses in group 1 (hydroxyethyl starch in normal saline solution) and 7 of 10 horses in group 2 (hydroxyethyl starch in lactated ringers solution) survived to hospital discharge. In addition, 8 horses in group 1 (hydroxyethyl starch in normal saline solution) and 9 horses in group 2 (hydroxyethyl starch in lactated ringers solution) developed complications during hospitalization. Complications observed included laminitis (n=4), DIC (1), jugular thrombosis (2), fever (5), nasogastric reflux (4), rectal prolapse (1), melena (3), peritonitis (2), endotoxemia (3), renal failure (1), colic pain (10), persistent tachycardia or tachypnea (7), incisional drainage or infection (3), and partial incisional dehiscence (1). Survival (*P*=0.650) and development of complications during hospitalization (*P*=1.000) did not differ significantly between treatment groups.

## Discussion

The results of the present study indicate that both hydroxyethyl starch formulations evaluated increase COP when used in the treatment of horses with low colloid oncotic pressure due to hypoproteinemia. Colloid supplementation improved COP approximately 20%, regardless of the formulation used. However, colloid treatment did not result in COP values within the equine normal range of 18-22 mmHg [7]. Venous pH was not affected by colloid administration. Therefore, it is presumed that type of colloid carrier fluid, normal saline versus lactate ringers solution, did not impact acid-base parameters in this setting.

Jones, P.A. and colleagues encountered similar results in measured COP after administration of 8-10ml/kg of 6% high molecular weight hydroxyethyl starch in 5 hypoproteinemic adult horses and 6 horses ≤1 year of age [8]. In that study, there was a significant increase in measured COP, a mean of 1.5 mmHg post-infusion (13%), that persisted for 24 hours and returned to baseline values at 48 hours [8]. Similar to the present study, the increase in measured COP, although significant, did not reach the normal values reported in healthy horses [8]. The limited increase in COP observed in response to both formulations of hydroxyethyl starch in the present study, in addition to volume expansion, is commonly observed in diseased populations of humans [9]. In healthy horses administered 10mL/kg hydroxyethyl starch, plasma volume expansion was increased approximately 17 or 19%, dependent on formulation, for up to 8 hours despite a sustained increase in COP for 24 hours [10]. The length of plasma volume treatment effect following hydroxyethyl starch administration in critically ill horses warrants further study.

While volume expansion effects are largely independent of hydroxyethyl starch solution formulation, other treatment effects are directly attributed to size, configuration, and carrier of these colloid solutions. Though not observed acutely in the present study, treatment with hydroxyethyl starch in saline vs balanced crystalloid solutions is more frequently associated with development of hyperchloremic metabolic acidosis in humans [4]. Hydroxyethyl starch suspended in balanced electrolyte solution also has been shown to preserve coagulation function, assessed by thromboelastography, better than those in saline in humans [11,12]. High molecular weight and molar substitution hydroxyethyl starch solutions, such as those evaluated in the present study and frequently used in treatment of horses with hypoproteinemia, have greater adverse effects on platelet function and other indices of coagulation than lower moleular weight and molar substitution formulations [10,13,14]. Future studies performed in the target population, critically ill horses, will complement those evaluating newer generations of hydroxyethyl starch solutions in healthy animals and further inform treatment decisions and monitoring in the diseased population.

In the present study, COP was increased following colloid treatment, but did not reach the normal range. It is unknown whether a higher dose would have resulted in a post-treatment COP value closer to the normal range or whether it is necessary to target a specific COP treatment goal in horses. Investigators evaluating 10, 20, and 40 mL/kg doses of low molecular weight and molar substitution hydroxyethyl starch solution in healthy horses did not observe linear increases in duration of COP elevation post infusion with dose, with significant elevation of COP observed for 24, 6, and 48 hours respectively [13]. A study in critically ill humans found that risk of mortality due to low COP could be eliminated by maintenance of a mean COP of >16 mmHg with colloid treatment, despite values of COP persistently below the normal range [15]. When combining the colloid groups evaluated in the present study, the mean pre-treatment COP value was 11 mmHg and post-treatment was 13.2 mmHg. Both pre- and post-colloid treatment mean COP values were below 16 mmHg. It is likely that the high case fatality rate (40%) observed was related to a combination of factors, namely illness severity and low COP.

In the present study, a limited number of clinical cases, with diverse disease etiology, were used to evaluate the effects of hydroxyethylstarch administration. Further, several horses required additional doses of colloid supplementation during hospitalization and the impact of such treatments is unknown. Serial measurements of COP, PCV, and venous blood gas parameters may have determined variability in parameter measurements over time and further distinguished beneficial effects from treatment. The outcome parameters evaluated were limited to COP, PCV, venous blood gas analysis, development of complications, and short term survival to hospital discharge. Inclusion of coagulation and long term survival outcomes may have demonstrated differences between groups.

## Conclusions

Based on the results of this preliminary clinical study, hydroxyethyl starch in lactated ringers solution was at least equivalent to hydroxyethyl starch in normal saline solution. Further study is needed to determine how these two products compare with regards to other outcome measures and complications. Evaluation of the relative effects of colloid formulation in horses with clinical disease is a future area of interest.

## Competing interests

None to declare.

## Authors' contributions

FB participated in the data analysis and draft of the manuscript, TK participated in the design of the study and data collection, and EH concieved of the study and participated in the design of the study, data collection and analysis, and draft of manuscript. All authors read and approved the final manuscript.

## Declarations

Funding for this study was provided by the Colorado State University Center for Companion Animal Studies. Intravenous starch preparations were provided by Abbott Animal Health, Chicago, IL, USA. Publications costs were provided by the Colorado State University Advances in Equine Health Fund.

This article has been published as part of *BMC Veterinary Research* Volume 10 Supplement 1, 2014: Selected articles from the Eleventh International Equine Colic Research Symposium. The full contents of the supplement are available online at http://www.biomedcentral.com/bmcvetres/supplements/10/S1. Publication of this supplement has been funded by The British Equine Veterinary Association.
